# Acoustic Analysis of Voluntary Coughs, Throat Clearings, and Induced Reflexive Coughs in a Healthy Population

**DOI:** 10.1007/s00455-023-10574-1

**Published:** 2023-05-28

**Authors:** Sofiana Mootassim-Billah, Jean Schoentgen, Marc De Bodt, Nicolas Roper, Antoine Digonnet, Mathilde Le Tensorer, Gwen Van Nuffelen, Dirk Van Gestel

**Affiliations:** 1 Department of Radiation Oncology, Speech Therapy, Institut Jules Bordet, Université Libre de Bruxelles, Brussels, Belgium; 2 Department of Biomechatronics, Université Libre de Bruxelles, Brussels, Belgium; 3 Department of Otolaryngology and Head and Neck Surgery, University Rehabilitation Center for Communication Disorders, Antwerp University Hospital, Antwerp, Belgium; 4 Department of Translational Neurosciences, Faculty of Medicine and Health Sciences, University of Antwerp, Antwerp, Belgium; 5 Department of Logopaedics and Audiological Sciences, Faculty of Medicine and Health Sciences, University of Ghent, Ghent, Belgium; 6 Department of Oto-Rhino-Laryngology and Head & Neck Surgery, Erasme Hospital, Université Libre de Bruxelles, Brussels, Belgium; 7 Department of Surgical Oncology, Head and Neck Surgery Unit, Institut Jules Bordet, Université Libre de Bruxelles, Brussels, Belgium; 8 Speech Therapy Unit, Institut Jules Bordet, Hôpital Universitaire de Bruxelles, Université Libre de Bruxelles, Brussels, Belgium; 9 Department of Radiation Oncology, Head and Neck Unit, Institut Jules Bordet, Université Libre de Bruxelles, Brussels, Belgium

**Keywords:** Cough assessment, Acoustic analysis, Dysphagia, Aspiration, Head and neck cancer, Chemoradiotherapy

## Abstract

Cough efficacy is considered a reliable predictor of the aspiration risk in head and neck cancer patients with radiation-associated dysphagia. Currently, coughing is assessed perceptually or aerodynamically. The goal of our research is to develop methods of acoustic cough analysis. In this study, we examined in a healthy population the acoustical differences between three protective maneuvers: voluntary cough, voluntary throat clearing, and induced reflexive cough. Forty healthy participants were included in this study. Voluntary cough, voluntary throat clearing, and reflexive cough samples were recorded and analyzed acoustically. Temporal acoustic features were the following: the slope and curvature of the amplitude contour, as well as the average, slope, and curvature of the sample entropy and kurtosis contours of the recorded signal. Spectral features were the relative energy in the frequency bands (0–400 Hz, 400–800 Hz, 800–1600 Hz, 1600 Hz-3200 Hz, > 3200 Hz) as well as the weighted spectral energy. Results showed that, compared to a voluntary cough, a throat clearing starts with a weaker onset pulse and involves oscillations from the onset to the offset (concave curvature of the amplitude contour, *p* < 0.05), lower average (*p* < 0.05), and slope (*p* < 0.05) as well as lower convex curvature (*p* < 0.05) of the kurtosis contour. An induced reflexive cough starts with a higher and briefer onset burst and includes higher frication noise (larger convexity of the curvature of the amplitude and kurtosis contours (*p* < 0.05)) compared to a voluntary cough. The conclusion is that voluntary coughs are acoustically significantly different from voluntary throat clearings and induced reflexive coughs.

## Introduction

Coughing is a protective mechanism preventing materials from entering the airways, especially in the context of dysphagia [1]. Deficient coughing before, during, or after swallowing is a clinical marker of dysphagia in head and neck cancer (HNC) patients [2, 3]. HNC patients treated with chemoradiotherapy are severely at-risk of radiation-associated dysphagia (RAD), that is impaired swallowing safety/efficiency following chemoradiotherapy [4–6]. Sensory deterioration in RAD may result in an ineffective or absent coughing possibly leading to aspiration of materials into the airways and consequently, aspiration pneumonia [7–10].

Coughing—defined as a deep inspiration followed by complete closure of the glottis, forced expiratory effort (compression), and finally opening of the glottis with expiration [11]—may be either voluntary or reflexive. A voluntary cough, originating in the cerebral cortex [12], is generally produced to clear residue or mucus in the upper laryngeal tract. A reflexive cough is elicited by contact of food, liquids, or chemicals with the true vocal folds, the false vocal cords, the aryepiglottic folds, or the upper tracheal areas [13].

Throat clearing is another protective maneuver frequently used voluntarily or reflexively by patients to clear the airways from mucus or laryngeal residue. Compared to a cough, a throat clearing starts without any prior inspiration and requires only partial glottal closure [1]. Even though a throat clearing is regularly assessed during a clinical swallowing evaluation, research on this maneuver is lacking.

As reported in our previous overview [14], established cough assessment includes aerodynamic, acoustic measures, and perceptual ratings. While examining reflexive coughs induced with a tussigen, cough airflow-related measures are currently regarded as reliable markers of dysphagia and aspiration in patients with neurological disorders [15–17]. However, these findings have not yet been corroborated in HNC patients with RAD. Moreover, aerodynamic equipment is not widely available in daily clinical practice, and it may interfere with an evaluation in a natural setting (during a meal, for instance) because of the presence of a pipe or a facemask.

Consequently, most clinicians (e.g., speech therapists, neurologists, and otorhinolaryngologists) rely on an auditory assessment of coughing during a clinical swallowing evaluation. A major issue with auditory assessment is that the inter-rater agreement is low [18], irrespective of the number of years of dysphagia management expertise or participation in training sessions on perceptual cough assessments [16, 18, 19]. One possible cause of the difficulty in auditorily reliably identifying markers of dysphagia and aspiration may be due to the disagreement among professionals regarding the terminology of the different maneuvers [18]. Also, cough-related auditory descriptors are generally not clearly defined by raters and not related to objective features [18–21]. Indeed, Laciuga et al. have shown that the auditory discrimination between a cough and a throat clearing may be unclear [18]. The cough strength and the cough quality (e.g., effortful, breathy, strained) are also inconsistently rated.

Regarding acoustic analysis, cough sounds have been found to reliably detect respiratory diseases such as Covid-19, chronic obstructive pulmonary disease, asthma, pneumonia, lower and upper respiratory tract diseases, croup, or bronchiolitis [22–24]. An acoustic cough emission is usually defined as a transient signal comprising three sequential phases: a burst/release, followed by a “fricated” fragment (due to turbulent airflow), and a non-mandatory “voiced” fragment [25, 26]. This academic view is inspired by the analogy between a cough sound and a glottal stop—a consonantal sound used in many spoken languages, produced by obstructing the airflow at the glottis followed by a release [27]. Basing their research on this three-phase model, numerous studies have found acoustic cough-related features for automatic distinction between some respiratory diseases [24, 28–32]. However, few relationships between acoustic cough features and perceptual ratings have been found [20, 21]. Also, no reliable acoustic cough-related features have been reported yet for assessing RAD in HNC patients, nor for assessing dysphagia and/or aspiration in other populations [33–36].

Conventional software for voice and speech analysis is not appropriate because of the transient nature of the cough signal. Indeed, the assessment of voice quality is based on sustained voiced speech sounds, selected for reasons of technical feasibility and ease of reproducibility of the analysis. Therefore, an analysis method considering the transient nature of the cough signal may improve the quality of acoustic cough analysis in RAD. Such a method should rely on objective features, would be easily implementable in daily clinical practice and compatible with a swallowing evaluation in a natural setting.

The overall goal of our research is to develop appropriate methods of acoustic cough analysis with a view to identifying acoustic features as possible markers of swallowing impairments in HNC patients with RAD. In this study, we examined in a healthy population the acoustical differences between three protective maneuvers: voluntary cough, voluntary throat clearing, and induced reflexive cough.

## Materials and Methods

### Participants

Forty healthy individuals participated in this study, the inclusion criterium was participants aged 18 years minimum. Exclusion criteria were (1) a history of head and neck cancer, (2) dysphagia (according to the Yale Swallow Protocol [37]), (3) dysphonia (G > 0 on GRBAS-I scale [38]), (4) a history of smoking within less than one year, and (5) an acute or chronic respiratory disease (e.g., chronic obstructive pulmonary disease or asthma).

### Recordings

**Fig. 1:**
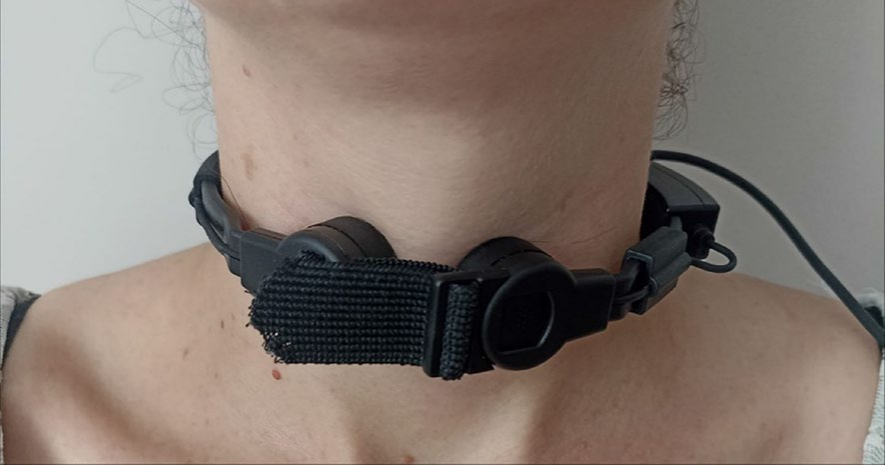
Illustration of the skin-contact microphone

All participants were recorded at the Jules Bordet Institute in Brussels. Participants were asked to produce five voluntary coughs, five voluntary throat clearings, and a minimum of two induced reflexive coughs. Participants were seated in an audiometric booth. The recordings were made using simultaneously a skin-contact microphone and a professional quality acoustic free-standing microphone. The skin-contact microphone was a Albrecht AE 38 S2 (Fig. 1), validated and found reliable for recordings in a natural setting [39]. The free-standing microphone was a AKG Perception 420 Omnidirectional, fixed to a flex arm fastened to a table facing the participants, placed at 40 cm and at an angle of approximately 45 degrees to the right of the mouth of the participant. A metallic anti-pop filter was placed in front of the microphone to prevent the exhaled air hitting the microphone, but also to ease disinfection with wipes. Intensity (in dB) of voluntary coughs and throat clearings were measured with an external sound level meter Bruel & Kjaer 2236 placed at 40 cm and at an angle of approximately 45 degrees to the right of the mouth of the participant, both for reasons of hygiene and to avoid the air hitting the microphone. All participants were instructed to remain motionless during voluntary maneuvers. For voluntary coughing, each participant was verbally instructed (in French) as follows: “Take a maximal breath and cough as if you have something stuck in your throat.” For voluntary throat clearing, the verbal instruction was “Clear your throat as if you have something stuck in your throat.” Participants producing a voluntary cough rather than a throat clearing following this instruction were given a demonstration by the experimenter.

**Fig. 2:**
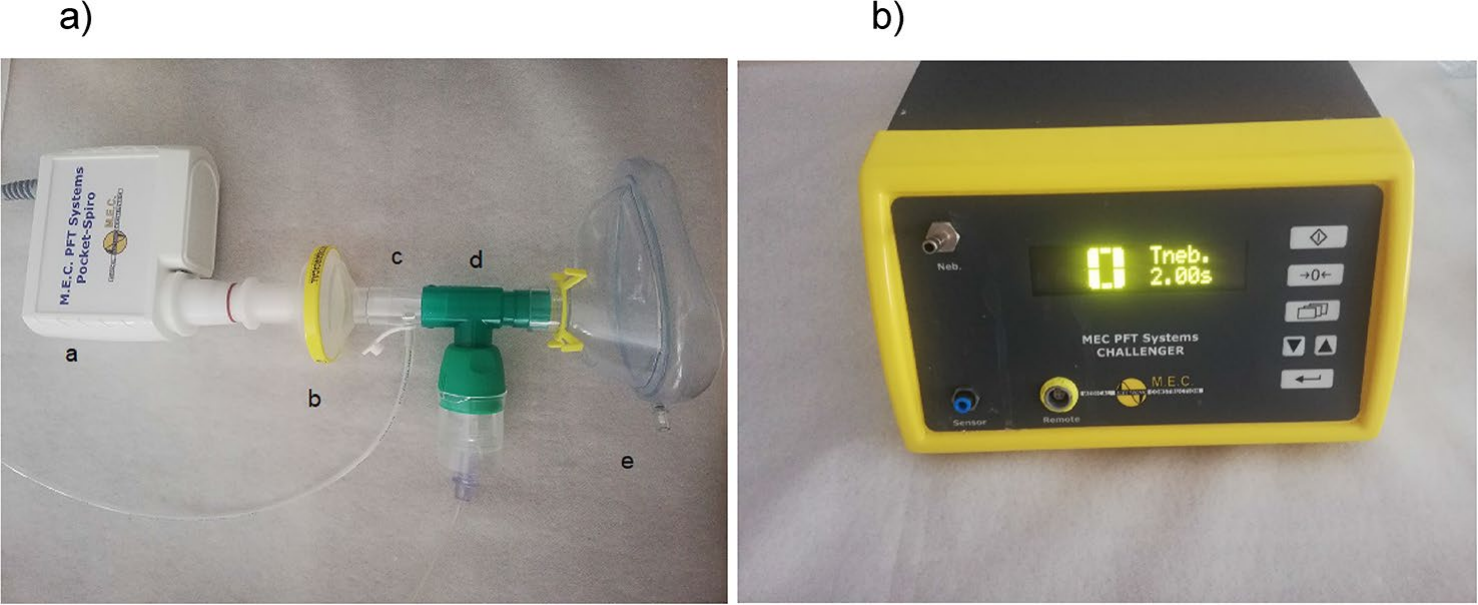
**a** Illustration of the aerodynamic equipment: a. spirometer, b. antibacterial filter, c. differential pressure transducer with a one-way inspiratory valve, d. t-piece containing citric acid, e. facemask. **b** Illustration of the nebulizer that delivers citric acid during a 2-s inspiration

Cough induction was performed with an anesthesia face mask connected to a spirometer Pocket-Spiro USB (Medical Electronic Construction, Belgium) and a differential pressure transducer with a one-way inspiratory valve for nebulizer connection (Fig. 2). The nebulizer delivered citric acid during a 2-s inspiration. Each participant completed a maximum of 5 challenges of concentrations of citric acid: saline, 30 mM or 5.8 mg/ml citric acid, 100 mM or 19.2 mg/ml citric acid, 300 mM or 58 mg/ml citric acid, and 1000 mM or 192 mg/ml citric acid as described in Janssens et al. [40]. To avoid tachyphylaxis (a decreased response to repeated stimulation), concentrations of citric acid were delivered incrementally, and all inter-trial intervals lasted for a minimum of 60 s. Reflexive coughs in response to the challenges were measured to define the lowest concentration at which 2 or more successive coughs (C2 threshold) are triggered after one single inspiration. For cough induction only, participants were divided in two groups. The first group (*N* = 20) was directly told “try not to cough” (the suppressed reflexive cough method). The second group (*N* = 20) was initially instructed “cough if you need to” (urge-to-cough method). After recording 2 successive coughs according to the urge-to-cough method, this group received incremental citric acid concentrations from the cough threshold now with the instruction “try not to cough.” This enabled recording induced reflexive coughs with regard to different methods. Only the skin-contact microphone was suitable for recording induced reflexive coughs because of the presence of the anesthesia facemask. Therefore, measuring intensity in dB with the external sound level meter was not possible.

As recommended by the Union of European Phoniatricians’ guidelines during the Covid-19 pandemic, investigators wore a protective visor for face and eye protection, a surgical face mask, a single-use protective gown, and a single use cap. A time interval of ten minutes between participants was scheduled for purifying and sterilizing the room with a Hextio Radic8 device and for cleaning all surfaces and equipment.

Cough samples were recorded with an HP ProBook computer (Hew1ett-Packard Company, USA) using the computer program PRAAT and the pre-amplifier 2 channel interface Presonus Audiobox USB 96 Audio, with a sampling frequency of 44.1 kHz. All recorded signals were analyzed with a software developed for the purpose of this study and written in the Python Programming Language.

A statistical software package (IBM SPSS Statistics 28) was used to obtain descriptive statistics of voluntary coughs, voluntary throat clearings, and induced reflexive coughs.

### Segmentation

Cough samples were segmented manually into single coughs leaving silent intervals before and after. The segmentation by hand of a single cough from its preceding and succeeding silent intervals is difficult because the offset of a single cough is drawn out without a well-defined boundary. A subsequent automatic segmentation was therefore carried out via the signal contour by assigning to the onset the first contour sample and to the offset the last contour sample the value of which is larger than − 30 dB with regard to the signal contour maximum. Before analysis, the segmented cough signals were normalized so that the average signal energy of the segmented recording is equal to one.

### Spectral Analysis

Cough signals are transient signals (average duration of 0.3 s), which are therefore unsatisfactorily represented by spectrograms. Spectrograms target sustained sounds during which the properties of the signal barely change. Indeed, a majority of sounds of biological origin, including speech sounds, have quasi-stationary frames which are connected by transitions. On the contrary, the cough signal evolves rapidly and incessantly over a short duration. We therefore focused on a smaller number of frequency intervals, the energies, and frequencies of which are reported band by band. Indeed, lowering the frequency resolution of the spectral analysis increases its temporal resolution.

The signals were decomposed into constituent signals via a filter bank that is based on the discrete cosine transform (DCT). The frequency boundaries were equal to 400 Hz, 800 Hz, 1600 Hz, and 3200 Hz. The difference between the discrete cosine and discrete Fourier transforms is that the former periodically extends the analyzed signal by pivoting the signal with regard to its onset and offset so that the periodically extended signal is even. The juxtaposition of a slow and low-amplitude offset with a rapid and high-amplitude onset is thus avoided, as well as the ensuing spectral artifacts. The decomposition of the cough signal by means of a DCT is exact, that is, the sum of the band-filtered signals as well as their signal energies is equal to the original cough signal and its energy [41].

The spectral features were the relative signal energies in the bands (0 Hz–400 Hz), (400 Hz–800 Hz), (800 Hz–1600 Hz), (1600 Hz–3200 Hz) as well as in the interval between 3200 Hz and half the sampling frequency. The typical frequency in each band is estimated via the number of unidirectional zero crossings. The per-band frequencies is weighted by the relative band energies and summed. The weighted sum is an approximation of the spectral centroid that subdivides the signal spectrum into two halves that have equal energies.

### Temporal Analysis

The temporal analysis involves the evolution with time of the contours of the cough signal amplitude, the sample entropy as well as the kurtosis.

The amplitude contour reports the relative strength of the cough signal. Because of the normalization, the average amplitude is equal to one. Normalization compensates for the influence of the pre-amplifier gain or the microphone position on the cough signal features. The intensity in dB of the cough signal was reported via an independent sound level meter instead.

The contour of the sample entropy reports the degree of randomness. The sample entropy enables segregating analysis frames according to whether they report turbulence noise or locally periodic oscillations because turbulence noise is expected to be less predictable than locally periodic oscillations [42].

The kurtosis contour reports the impulsive quality of the signal samples. The kurtosis may be interpreted in terms of the peakedness of the histogram of the sample values in the analysis frame. Sample histograms that are between normal and uniform have kurtosis values between three and zero. Histograms, the peakedness of which is stronger than normal have kurtosis values larger than three. Burst-like onsets are therefore expected to have larger kurtosis values than turbulence noise or oscillations [43].

The shape of the contours of the cough amplitude, sample entropy, and kurtosis is described by means of the first three DCT coefficients. Inspecting the pattern of the first three co-sinusoidal basis functions shows that the first coefficient was the contour average. The second coefficient describes the contour slope. A positive coefficient value indicates a slope that is decreasing with time. The third coefficient reports the contour curvature. A positive coefficient value indicates a downward–upward (convex) curvature and a negative value indicates an upward–downward (concave) curvature of the contour with regard to the horizontal.

## Results

Forty healthy individuals, including 25 women and 15 men, have participated in this study. Participants were recruited on a voluntary basis among hospital staff and external hospital parties. The average age of the participants was 40.1 ± 10.3 years (range, 24 to 65 years). The average age for the women was 39,5 ± 11,1 (range, 24 to 59) and the average age for the male subjects was 41,1 ± 12,5 (range, 29 to 65).

All samples of voluntary coughs and voluntary throat clearings obtained in this study were considered suitable for analysis. Of the 20 participants instructed to attempt to suppress the reflexive cough, only one did not produce any induced reflexive cough (lack of response). Of the 20 participants that received two different injunctions before cough inducing, one recording was discarded due to an error of manipulation. Thirteen participants produced reflexive coughs following the suppressed reflexive cough method (seven participants lacked a cough response). Because the first two consecutive single coughs after one single inhalation of citric acid were considered for analysis, the total number of single coughs analyzed was 102 (urge-to-cough instruction *N* = 38 and suppressed reflexive cough instruction *N* = 64).

The medians, quartiles, minima, maxima, and bootstrapped confidence intervals for the medians (95%) of the acoustic features recorded with the free-standing and the skin-contact microphones were analyzed and a statistical comparison by means of non-parametric Wilcoxon or Mann–Whitney U tests are reported in Tables 1, 2, 3, 4, 5, 6, 7, 8.

**Table 1:** Medians, quartiles, minima, maxima, and confidence intervals (95%) for the medians of intensity, signal length, amplitude, sample entropy, and kurtosis as well as the statistical significance of the difference between a voluntary cough and a throat clearing with a free-standing microphone

	Voluntary cough (free-standing mic) (*N* = 200)						Throat clearings (free-standing mic) (*N* = 200)						Wilcoxon tests
	Q1	Med	Q3	Min	Max	Med. 95%CI	Q1	Med	Q3	Min	Max	Med 95%CI	
Intensity (dB)—external sound level meter													
	93	97	101	85	110	96 98	88	92	96	81	104	91 93	***p* < 0.001**
Length (sec)													
	0.305	0.388	0.473	0.188	0.970	0.370 0.400	0.380	0.458	0.526	0.137	1.012	0.444 0.475	***p* < 0.001**
Amplitude													
Slope	0.139	0.296	0.439	− 0.582	0.724	0.262 0.369	0.150	0.242	0.360	− 0.542	0.761	0.216 0.265	***p* = 0.025**
Curvature	− 0.134	0.024	0.165	− 0.652	0.349	− 0.013 0.066	− 0.379	− 0.266	− 0.147	− 0.761	− 0.428	− 0.288 − 0.230	***p* < 0.001**
Sample entropy													
Average	0.540	0.665	0.821	0.349	1.187	0.622 0.709	0.312	0.412	0.597	0.148	0.921	0.384 0.437	***p* < 0.001**
Slope	0.027	0.103	0.176	− 0.209	0.317	0.085 0.120	− 0.006	0.051	0.104	− 0.172	0.256	0.040 0.068	***p* < 0.001**
Curvature	− 0.155	− 0.102	− 0.049	− 0.318	0.104	− 0.115 − 0.085	− 0.035	0.004	0.043	− 0.227	0.160	− 0.003 0.012	***p* < 0.001**
Kurtosis													
Average	3.053	3.298	3.526	2.406	4.524	3.231 3.333	2.587	2.820	3.111	2.091	5.518	2.742 2.895	***p* < 0.001**
Slope	0.176	0.330	0.484	− 0.304	1.190	0.295 0.373	0.092	0.189	0.319	− 0.251	0.939	0.170 0.226	***p* < 0.001**
Curvature	0.108	0.254	0.386	− 0.611	0.991	0.216 0.284	0.109	0.219	0.322	− 0.372	2.299	0.191 0.239	*p* = 0.171

Bold indicates statistically significant difference *p* < 0.05

**Table 2:** Medians, quartiles, minima, maxima, and confidence intervals (95%) for the medians of the relative energy in each frequency band and spectral centroid as well as the statistical significance of the difference between a voluntary cough and a throat clearing with a free- standing microphone

	Voluntary cough (free-standing mic) (*N* = 200)						Throat clearings (free-standing mic) (*N *= 200)						Wilcoxon tests
	Q1	Med	Q3	Min	Max	Med 95%CI	Q1	Med	Q3	Min	Max	Med 95%CI	
< 400 Hz	0.229	0.431	0.625	0.016	0.902	0.393 0.493	0.533	0.770	0.911	0.156	0.982	0.716 0.841	***p* < 0.001**
400–800 Hz	0.060	0.102	0.172	0.013	0.695	0.089 0.116	0.023	0.056	0.127	0.004	0.488	0.048 0.072	***p* < 0.001**
800–1600 Hz	0.050	0.108	0.194	0.013	0.739	0.086 0.119	0.011	0.040	0.096	0.001	0.530	0.027 0.047	***p* < 0.001**
1600–3200 Hz	0.079	0.149	0.246	0.019	0.538	0.125 0.165	0.026	0.051	0.105	0.003	0.513	0.042 0.064	***p* < 0.001**
> 3200 Hz	0.054	0.091	0.167	0.009	0.544	0.079 0.115	0.006	0.016	0.058	0.001	0.419	0.013 0.023	***p* < 0.001**
Weighted freq. (Hz)	868	1342	1838	477	3968	1246 1455	357	515	934	182	2578	480 612	***p* < 0.001**

Bold indicates statistically significant difference *p* < 0.05

**Table 3:** Medians, quartiles, minima, maxima, and confidence intervals (95%) for the medians of signal length, amplitude, sample entropy, and kurtosis as well as the statistical significance of the difference between a voluntary cough and a throat clearing with a skin-contact microphone

	Voluntary cough (skin-contact mic) (*N *= 200)						Throat clearings (skin-contact mic) (*N *= 200)						Wilcoxon tests
	Q1	Med	Q3	Min	Max	Med 95%CI	Q1	Med	Q3	Min	Max	Med 95%CI	
Length (sec)													
	0.267	0.332	0.395	0.054	0.612	0.317 0.347	0.296	0.357	0.428	0.074	0.834	0.340 0.383	***p* < 0.001**
Amplitude													
Slope	− 0.027	0.226	0.383	− 0.422	0.738	0.174 0.274	0.117	0.240	0.355	− 0.556	0.672	0.216 0.261	***p* = 0.037**
Curvature	− 0.036	0.205	0.355	− 0.530	0.640	0.171 0.260	− 0.309	− 0.215	− 0.115	− 0.721	0.392	− 0.231 − 0.193	***p* < 0.001**
Sample entropy													
Average	0.244	0.293	0.336	0.094	0.880	0.277 0.304	0.190	0.223	0.267	0.104	0.690	0.211 0.234	***p* < 0.001**
Slope	− 0.026	0.005	0.028	− 0.178	0.098	− 0.006 0.010	− 0.014	0.003	0.018	− 0.177	0.103	0.000 0.007	*p* = 0.594
Curvature	− 0.069	− 0.053	− 0.028	− 0.367	0.050	− 0.058 − 0.047	− 0.016	− 0.004	0.006	− 0.130	0.079	− 0.007 − 0.002	***p* < 0.001**
Kurtosis													
Average	3.225	3.581	3.937	2.441	14.60	3.480 3.700	3.061	3.409	3.738	1.972	5.970	3.326 3.478	***p* < 0.001**
Slope	0.007	0.242	0.518	− 0.732	1.720	0.191 0.300	− 1.578	0.073	0.235	− 1.404	1.011	0.004 1.142	***p* < 0.001**
Curvature	0.225	0.435	0.691	− 1.717	2.162	0.380 0.495	0.188	0.314	0.514	− 1.570	1.919	0.284 3.373	***p* = 0.001**

Bold indicates statistically significant difference *p* < 0.05

**Table 4:** Medians, quartiles, minima, maxima, and confidence intervals (95%) for the medians of the relative energy in each frequency band and spectral centroid as well as the statistical significance of the difference between a voluntary cough and a throat clearing with a skin-contact microphone

	Voluntary cough (SCmic) (*N *= 200)						Throat clearings (SCmic) (*N *= 200)						Wilcoxon tests
	Q1	Med	Q3	Min	Max	Med 95%CI	Q1	Med	Q3	Min	Max	Med 95%CI	
< 400 Hz	0.286	0.441	0.585	0.010	0.961	0.396 0.490	0.316	0.473	0.608	0.032	0.951	0.424 0.507	*p* = 0.083
400–800 Hz	0.326	0.446	0.570	0.017	0.919	0.412 0.473	0.228	0.457	0.570	0.019	0.899	0.419 0.489	*p* = 0.253
800–1600 Hz	0.035	0.065	0.112	0.004	0.641	0.055 0.074	0.024	0.041	0.078	0.004	0.667	0.035 0.047	***p* < 0.001**
1600–3200 Hz	0.003	0.007	0.012	0.000	0.072	0.006 0.008	0.002	0.004	0.009	0.000	0.266	0.003 0.005	***p* < 0.001**
> 3200 Hz	0.000	0.000	0.001	0.000	0.011	0.000 0.000	0.000	0.000	0.000	0.000	0.013	0.000 0.000	***p* = 0.007**
Weighted freq. (Hz)	430	504	597	307	936	483 518	406	469	533	265	945	456 487	***p* < 0.001**

Bold indicates statistically significant difference *p* < 0.05

**Table 5:** Medians, quartiles, minima, maxima, and confidence intervals (95%) for the medians of signal length, amplitude, sample entropy, and kurtosis as well as the statistical significance of the difference between an induced reflexive cough with the urge-to-cough method and an induced reflexive cough with the suppressed reflexive cough method (skin-contact microphone)

	Urge-to-cough method (skin-contact mic) (*N *= 38)						Suppressed reflexive cough method (skin-contact mic) (*N *= 64)						Mann–Whitney U tests
	Q1	Med	Q3	Min	Max	Med 95%CI	Q1	Med	Q3	Min	Max	Med 95%CI	
Length (sec)													
	0.267	0.334	0.364	0.168	0.752	0.285 0.353	0.258	0.304	0.354	0.134	0.902	0.286 0.321	*p* = 0.311
Amplitude													
Slope	− 0.051	0.151	0.530	− 0.325	0.739	0.059 0.342	0.027	0.267	0.521	− 0.511	0.751	0.154 0.445	*p* = 0.414
Curvature	0.066	0.264	0.475	− 0.521	0.601	0.201 0.403	0.206	0.369	0.509	− 0.497	0.707	0.304 0.441	*p* = 0.106
Sample entropy													
Average	0.311	0.345	0.382	0.226	0.522	0.324 0.365	0.292	0.347	0.383	0.166	0.474	0.320 0.366	*p* = 0.798
Slope	− 0.024	0.000	0.024	− 0.093	0.057	− 0.009 0.018	− 0.024	− 0.007	0.018	− 0.110	0.071	− 0.011 0.000	*p* = 0.341
Curvature	− 0.083	− 0.075	− 0.055	− 0.165	0.009	− 0.078 − 0.061	− 0.082	− 0.065	− 0.046	− 0.133	− 0.006	− 0.074 − 0.058	*p* = 0.376
Kurtosis													
Average	3.466	3.839	4.336	2.923	4.910	3.625 4.010	3.460	3.390	4.253	3.016	6.671	3.656 4.048	*p* = 0.857
Slope	− 0.063	0.103	0.293	− 1.163	1.357	0.191 0.219	− 0.207	0.130	0.412	− 1.717	1.937	− 0.059 0.273	*p* = 0.923
Curvature	0.379	0.693	1.033	− 0.023	1.504	0.503 0.889	0.355	0.513	0.805	− 0.549	1.622	0.409 0.650	*p* = 0.099

**Table 6:** Medians, quartiles, minima, maxima, and confidence intervals (95%) for the medians of the relative energy in each frequency band and spectral centroid as well as the statistical significance of the difference between an induced reflexive cough with the urge-to-cough method and an induced reflexive cough with the suppressed reflexive cough method (skin-contact microphone)

	Urge-to-cough method (skin-contact mic) (*N *= 38)						Suppressed reflexive cough method (skin-contact mic) (*N *= 64)						Mann–Whitney U tests
	Q1	Med	Q3	Min	Max	Med 95%CI	Q1	Med	Q3	Min	Max	Med 95%CI	
< 400 Hz	0.194	0.338	0.532	0.079	0.855	0.241 0.468	0.239	0.386	0.632	0.089	0.914	0.295 0.518	*p* = 0.183
400–800 Hz	0.327	0.499	0.636	0.100	0.780	0.360 0.576	0.240	0.416	0.596	0.078	0.783	0.322 0.505	*p* = 0.130
800–1600 Hz	0.052	0.087	0.169	0.014	0.527	0.057 0.136	0.062	0.092	0.179	0.006	0.411	0.082 0.119	*p* = 0.511
1600–3200 Hz	0.005	0.010	0.024	0.002	0.066	0.008 0.016	0.004	0.009	0.019	0.001	0.116	0.007 0.013	*p* = 0.637
> 3200 Hz	0.000	0.000	0.001	0.000	0.013	0.000 0.001	0.000	0.000	0.001	0.000	0.005	0.000 0.001	*p* = 0.635
Weighted freq. (Hz)	472	547	604	280	1021	508 591	432	527	618	286	831	481 571	*p* = 0.476

**Table 7:** Medians, quartiles, minima, maxima, and confidence intervals (95%) for the medians of signal length, amplitude, sample entropy, and kurtosis as well as the statistical significance of the difference between a voluntary cough and an induced reflexive cough (both methods combined) with the skin-contact microphone

	Voluntary cough (skin-contact mic) (*N *= 200)						Induced reflexive cough (skin-contact mic) (*N *= 102)						Mann–Whitney U tests
	Q1	Med	Q3	Min	Max	Med 95%CI	Q1	Med	Q3	Min	Max	Med 95%CI	
Length (sec)													
	0.267	0.332	0.395	0.054	0.612	0.317 0.347	0.261	0.314	0.359	0.134	0.902	0.292 0.332	*p* = 0.197
Amplitude													
Slope	− 0.027	0.226	0.383	− 0.422	0.738	0.174 0.274	0.004	0.221	0.523	− 0.511	0.751	0.141 0.364	*p* = 0.202
Curvature	− 0.036	0.205	0.355	− 0.530	0.640	0.171 0.260	0.168	0.352	0.497	− 0.521	0.707	0.269 0.403	***p* < 0.001**
Sample entropy													
Average	0.244	0.293	0.336	0.094	0.880	0.277 0.304	0.298	0.346	0.382	0.166	0.522	0.328 0.359	***p* < 0.001**
Slope	− 0.026	0.005	0.028	− 0.178	0.098	− 0.006 0.010	− 0.023	− 0.005	− 0.020	− 0.110	0.071	− 0.009 0.001	*p* = 0.360
Curvature	− 0.069	− 0.053	− 0.028	− 0.367	0.050	− 0.058 − 0.047	− 0.082	− 0.068	− 0.047	− 0.165	0.009	− 0.075 − 0.060	***p* < 0.001**
Kurtosis													
Average	3.225	3.581	3.937	2.441	14.60	3.480 3.700	3.467	3.888	4.259	2.923	6.671	3.680 4.008	***p* < 0.001**
Slope	0.007	0.242	0.518	− 0.732	1.720	0.191 0.300	− 0.193	0.113	0.391	− 1.717	1.937	0.037 0.207	***p* = 0.008**
Curvature	0.225	0.435	0.691	− 1.717	2.162	0.380 0.495	0.355	0.556	0.878	− 0.549	1.622	0.489 0.678	***p* = 0.002**

Bold indicates statistically significant difference *p* < 0.05

**Table 8:** Medians, quartiles, minima, maxima, and confidence intervals (95%) for the medians of the relative energy in each frequency band and spectral centroid as well as the statistical significance of the difference between a voluntary cough and an induced reflexive cough (both methods combined) with the skin-contact microphone

	Voluntary cough (skin-contact mic) (*N *= 200)						Induced reflexive cough (skin-contact mic) (*N *= 102)						Mann–Whitney U tests
	Q1	Med	Q3	Min	Max	Med 95%CI	Q1	Med	Q3	Min	Max	Med 95%CI	
< 400 Hz	0.286	0.441	0.585	0.010	0.961	0.396 0.490	0.217	0.362	0.600	0.079	0.914	0.296 0.467	*p* = 0.230
400–800 Hz	0.326	0.446	0.570	0.017	0.919	0.412 0.473	0.269	0.438	0.607	0.078	0.783	0.372 0.519	*p* = 0.709
800–1600 Hz	0.035	0.065	0.112	0.004	0.641	0.055 0.074	0.057	0.089	0.177	0.006	0.527	0.079 0.106	***p* < 0.001**
1600–3200 Hz	0.003	0.007	0.012	0.000	0.072	0.006 0.008	0.005	0.009	0.020	0.001	0.116	0.008 0.013	***p* = 0.004**
> 3200 Hz	0.000	0.000	0.001	0.000	0.011	0.000 0.000	0.000	0.000	0.001	0.000	0.013	0.000 0.001	***p* = 0.021**
Weighted freq. (Hz)	430	504	597	307	936	483 518	442	534	506	280	1021	510 569	*p* = 0.084

Bold indicates statistically significant difference *p* < 0.05

### Voluntary Coughs

**Fig. 3:**
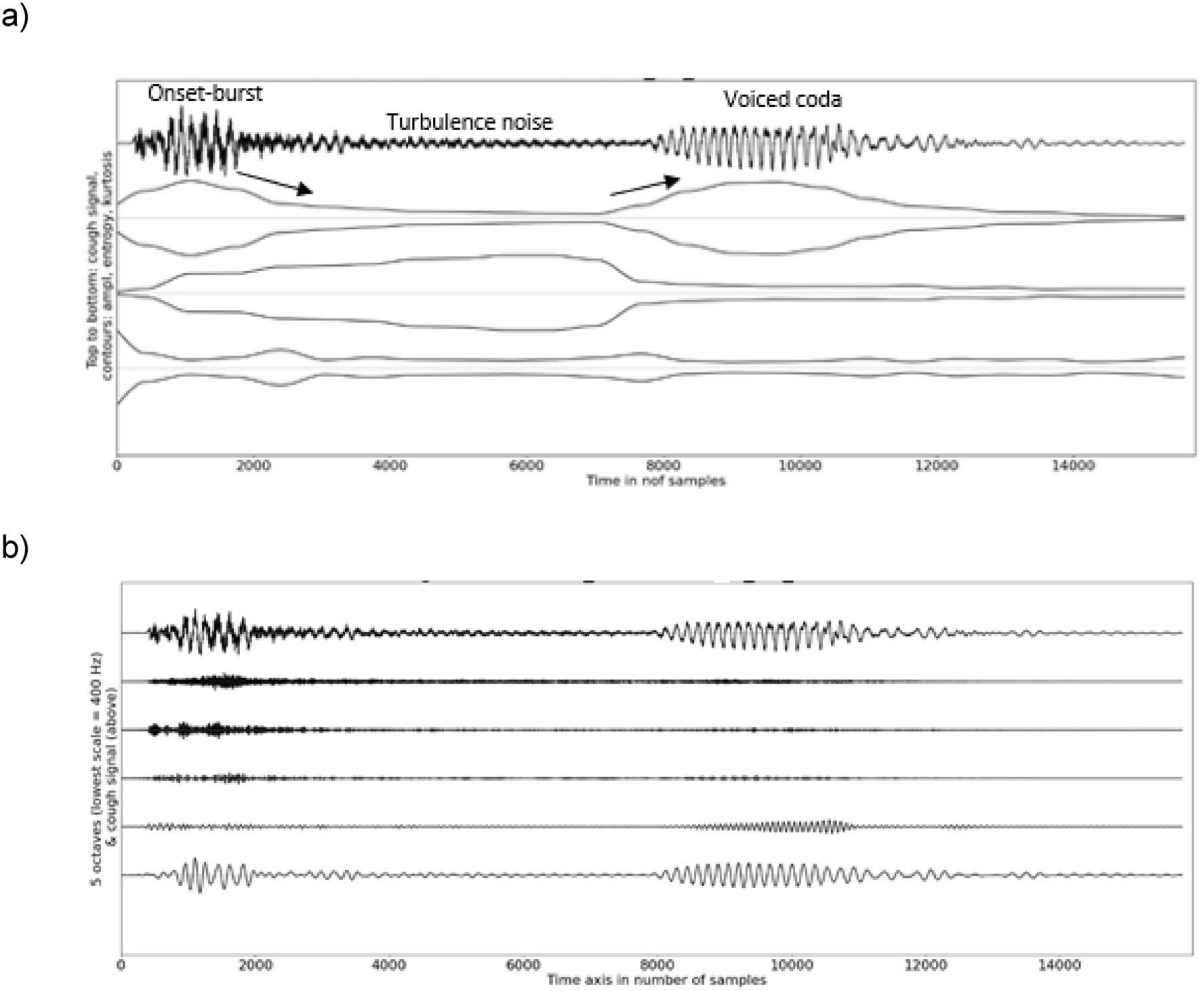
**a** Example of a graph illustrating the temporal analysis of a voluntary cough with a free-standing microphone, **b** example of a graph illustrating the spectral analysis of a voluntary cough with a free-standing microphone

**Fig. 4:**
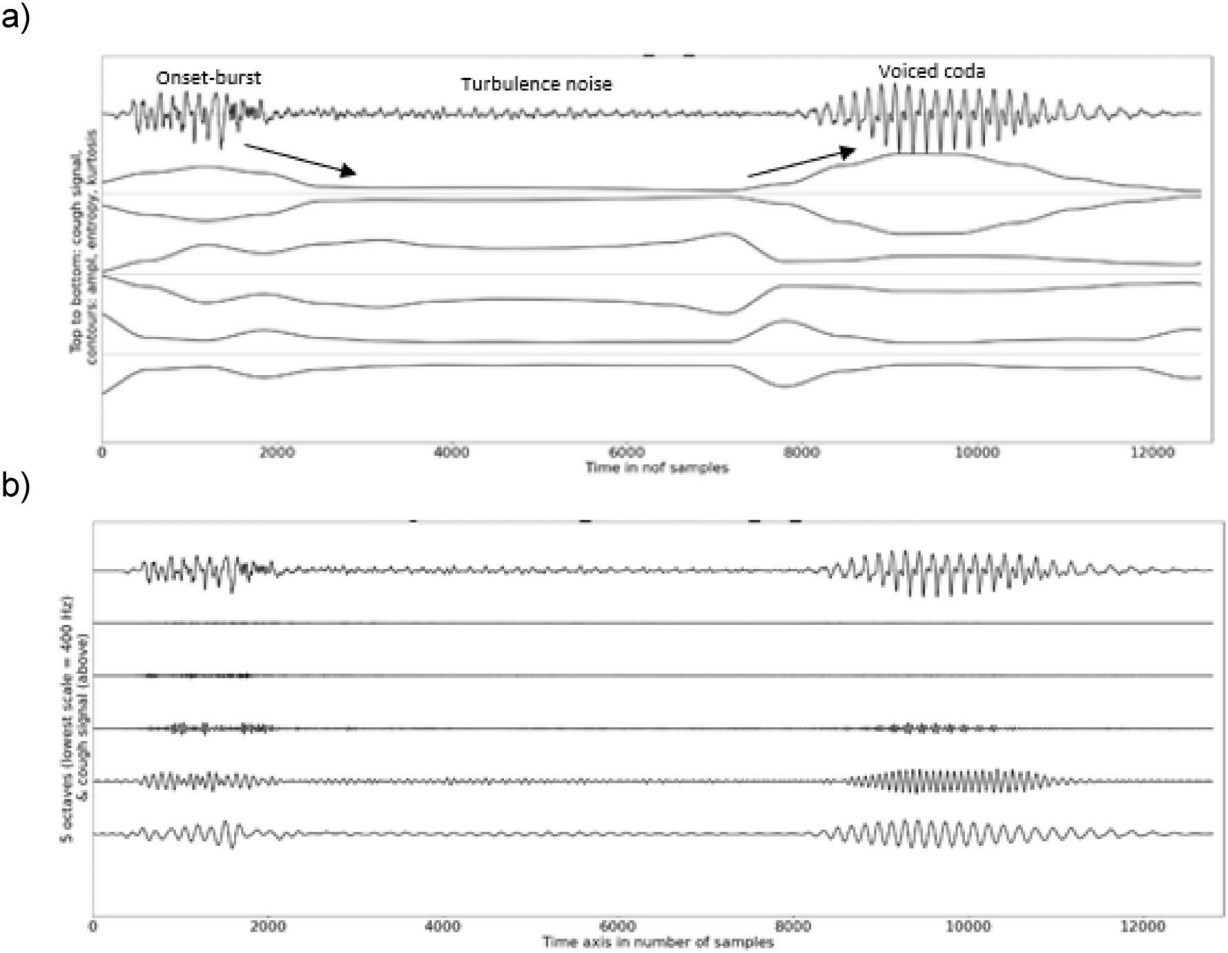
**a** Example of a graph illustrating the temporal analysis of a voluntary cough with a skin-contact microphone, **b** example of graph illustrating the spectral analysis of a voluntary cough with a skin-contact microphone

Analyses of voluntary coughs enabled to observe relevant patterns among temporal and spectral features (Tables 1, 2, 3, 4). Observed patterns were similar regardless of the microphone type (Figs. 3 and 4) and consistent between participants (compact confidence intervals of the medians). The amplitude contour of a voluntary cough was the largest at the onset and then decreased progressively to increase again towards the offset when the cough signal included a voiced coda (a non-mandatory voiced fragment). The onset was burst-like as shown by a large sample entropy and kurtosis and might, but must not, include low-frequency oscillations below 800 Hz, while the amplitude of the voiced coda and its offset were related to low-frequency energy exclusively. Indeed, we observed low-frequency oscillations below 800 Hz during the burst-like onset of a majority of voluntary cough signals and less frequently in the fricative fragment of the cough signal. The sample entropy, which is responsive to turbulence noise, displayed a concave contour as it increased progressively until mid-signal where it was stable before decreasing towards the voiced coda and its offset. The kurtosis was the highest (> 3) at the burst-onset of the signal.

The spectral centroid of voluntary coughs showed the spectrum of a cough signal recorded via the free-standing microphone to be more broadband than the same signal recorded via the skin-contact microphone, which underreports high-frequency components of the cough signal.

### Throat Clearings

**Fig. 5:**
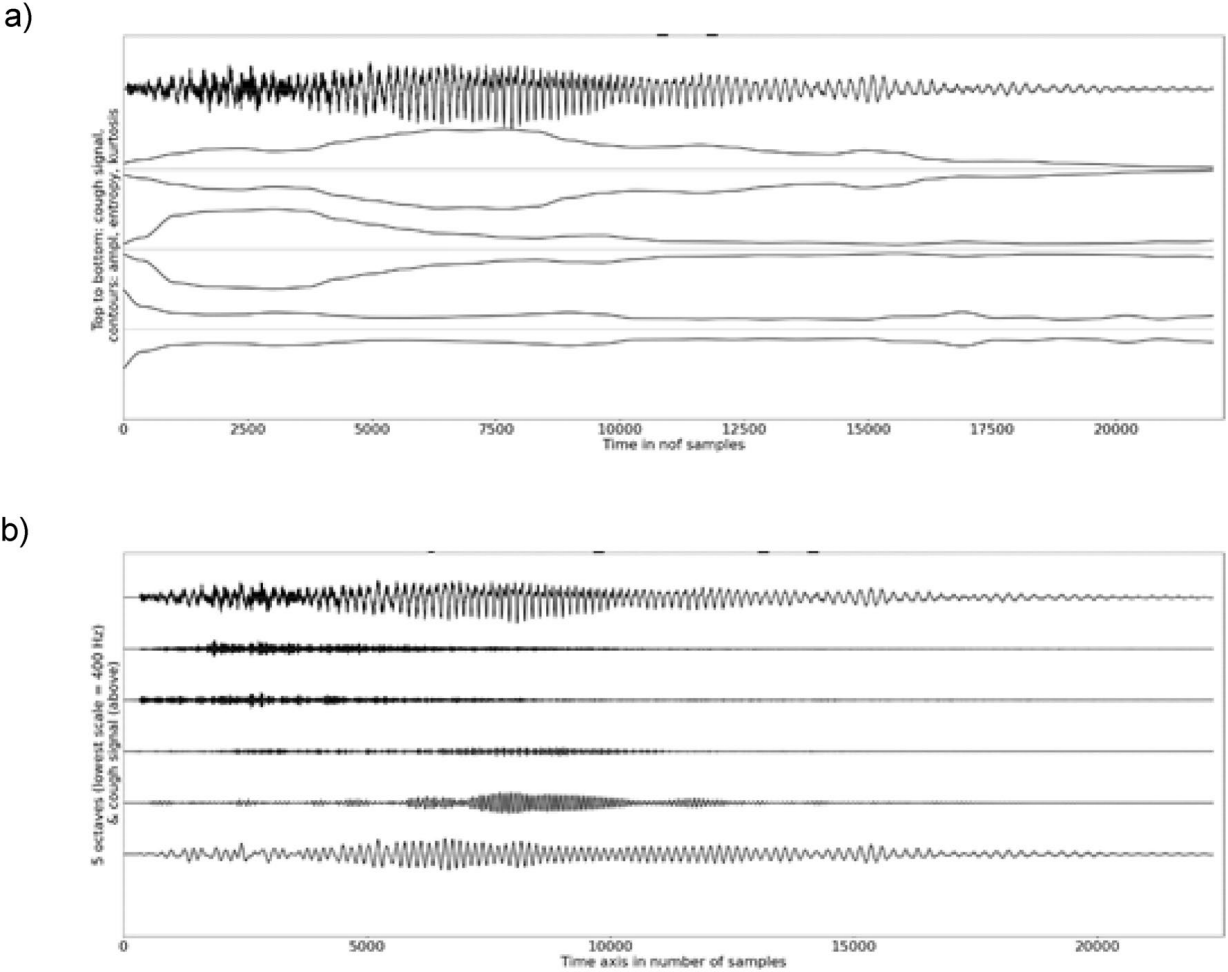
**a** Example of a graph illustrating the temporal analysis of a throat clearing with a free-standing microphone, **b** example of a graph illustrating the spectral analysis of a throat clearing with a free-standing microphone

**Fig. 6:**
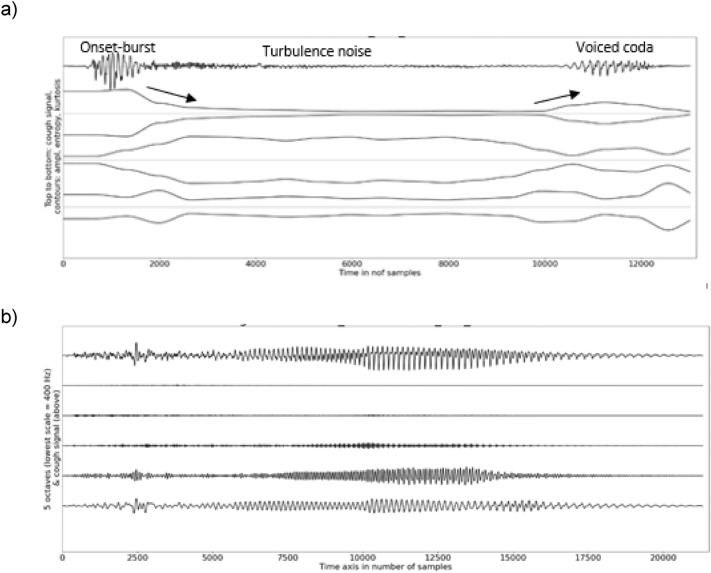
**a** Example of a graph illustrating the temporal analysis of a throat clearing with a skin-contact microphone, **b** example of a graph illustrating the spectral analysis of a throat clearing with a skin-contact microphone

The amplitude contour of a throat clearing was high all along the signal regardless of the transducer (Tables 1, 2, 3, 4). This was confirmed by a large relative energy below 800 Hz from the onset to the offset. A visual inspection showed throat clearings to start with a high kurtosis regardless of the microphone (Figs. 5–6). In the case of the free-standing acoustic microphone, the sample entropy was large until mid-signal, before decreasing progressively towards the offset. The sample entropy was less in the case of the skin-contact microphone. In addition, the relative energy in frequency bands higher than 800 Hz was very low in the case of the free-standing microphone and almost zero in the case of the skin-contact microphone. The spectral centroid was lower for signal recorded by means of the skin-contact microphone because this transducer underreports high-frequency spectral components. Compact confidence intervals of the medians suggested the temporal and spectral features to be consistent between participants regardless of the microphone type.

### Induced Reflexive Coughs

Induced reflexive coughs were recorded only with the skin-contact microphone because of the noise emitted by the nebulizer. In a first step, we compared the induced cough features obtained via the suppressed reflexive cough method (*N* = 64) with those obtained via the urge-to-cough method (*N* = 38) (Tables 5–6). Temporal and spectral acoustic cough features were not influenced by the choice of the urge-to-cough or suppressed reflexive cough method although it has been shown that this choice may significantly impact aerodynamic cough features [33, 44, 45]. Because of the lack of a significant statistical difference between the median values, the overlap of the confidence intervals of the medians, and the lack of differences between the histograms of the values obtained via both methods, we grouped the two types of recordings for analysis (Tables 7, 8).

**Fig. 7:**
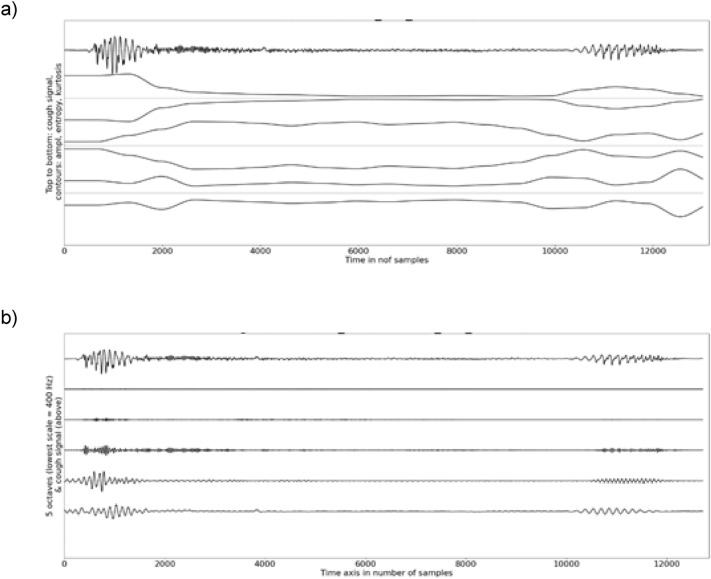
**a** Example of graph illustrating the temporal analysis of an induced reflexive cough with a skin-contact microphone, **b** example of graph illustrating the spectral analysis of an induced reflexive cough with a skin-contact microphone

A visual inspection showed reflexive cough contours to start with a large and short amplitude pulse, which is corroborated by high kurtosis at the onset (Fig. 7). A slighter visual re-increase of the relative amplitude and high kurtosis contours were noticeable when a voice coda was part of the cough signal. This was confirmed by a high spectral energy observed in the lower two frequency bands (below 1600 Hz) at the onset and in the voiced coda. The sample entropy contour displayed a concave curvature with low values at the onset and the offset but, large and stable values in the fricative fragment. A low degree of variability between participants was observed both for the temporal and spectral features (compact range of the confidence intervals of the medians).

We observed that the two consecutive single coughs within a reflexive cough bout (C2) to be distinguishable with regard to their acoustic feature values (Tables 9, 10). Indeed, cough #2 gave rise to statistically significant higher values of the amplitude contour slope (*p* = 0.006) and kurtosis contour slope (*p* = 0.008) as well as a higher spectral energy in the frequency band 400–800 Hz (*p* = 0.0019). In addition, the concavity of the sample entropy was higher for cough #1 than for cough #2 (*p* = 0.037).

**Table 9:** Medians, quartiles, minima, maxima, and confidence intervals (95%) for the medians of signal length, amplitude, sample entropy, and kurtosis as well as the statistical significance of the difference between cough #1 and cough #2 within induced reflexive coughs bouts (C2) recorded with the skin-contact microphone

		Cough #1 (induced reflexive cough) (skin-contact microphone) (*N *= 51)						Cough #2 (induced reflexive cough) (skin-contact microphone) (*N *= 51)						Wilcoxon tests
		Q1	Med	Q3	Min	Max	Med 95%CI	Q1	Med	Q3	Min	Max	Med 95%CI	
Length (sec)														
		0.285	0.319	0.364	0.175	0.902	0.295 0.348	0.251	0.295	0.350	0.134	0.612	0.267 0.327	*p* = 0.069
Amplitude														
Slope	− 0.018		0.157	0.397	− 0.325	0.665	0.069 0.269	0.015	0.458	0.590	− 0.511	0.751	0.156 0.547	***p* = 0.006**
Curvature	0.207		0.376	0.506	− 0.497	0.657	0.269 0.433	0.125	0.310	0.476	− 0.521	0.707	0.212 0.410	*p* = 0.512
Sample entropy														
Average	0.299		0.345	0.398	0.166	0.522	0.321 0.367	0.295	0.347	0.380	0.223	0.460	0.319 0.363	*p* = 0.353
Slope	− 0.017		− 0.006	0.016	− 0.065	0.041	− 0.009 0.000	− 0.041	− 0.001	0.031	− 0.110	0.071	− 0.011 0.016	*p* = 0.855
Curvature	− 0.090		− 0.072	− 0.053	− 0.165	− 0.008	− 0.077 − 0.062	− 0.080	− 0.062	− 0.045	− 0.134	0.009	− 0.075 − 0.056	***p* = 0.037**
Kurtosis														
Average	3.476		3.772	4.158	3.033	5.180	3.628 4.005	3.378	3.985	4.322	2.923	6.671	3.738 4.216	*p* = 0.456
Slope	− 0.250		0.021	0.272	− 1.163	0.944	− 0.191 0.119	0.005	0.232	0.673	− 1.717	1.937	0.106 0.370	***p* = 0.008**
Curvature	0.396		0.692	0.932	− 0.549	1.525	0.539 0.837	0.263	0.494	0.721	− 0.401	1.622	0.392 0.662	*p* = 0.067

Bold indicates statistically significant difference *p* < 0.05

**Table 10:** Medians, quartiles, minima, maxima, and confidence intervals (95%) for the medians of the relative energy in each frequency band and spectral centroid as well as the statistical significance of the difference between cough #1 and cough #2 within induced reflexive coughs bouts (C2) recorded with the skin-contact microphone

	Cough #1 (induced reflexive cough) (skin-contact microphone) (*N *= 51)						Cough #2 (induced reflexive cough) (skin-contact microphone) (*N *= 51)							Wilcoxon tests
	Q1	Med	Q3	Min	Max	Med 95%CI	Q1	Med		Q3	Min	Max	Med 95%CI	
< 400 Hz	0.207	0.413	0.637	0.079	0.914	0.295 0.511	0.220		0.329	0.544	0.086	0.815	0.280 0.429	*p* = 0.161
400-800 Hz	0.246	0.393	0.598	0.078	0.783	0.322 0.489	0.281		0.494	0.631	0.100	0.761	0.376 0.575	***p* = 0.019**
800-1600 Hz	0.056	0.086	0.166	0.006	0.527	0.072 0.130	0.060		0.093	0.179	0.014	0.411	0.070 0.119	*p* = 0.743
1600-3200 Hz	0.004	0.009	0.022	0.001	0.116	0.007 0.014	0.005		0.010	0.019	0.001	0.066	0.008 0.014	*p* = 0.493
> 3200 Hz	0.000	0.000	0.001	0.000	0.005	0.000 0.001	0.000		0.000	0.001	0.000	0.013	0.000 0.001	*p* = 0.587
Weighted freq. (Hz)	439	530	614	286	1021	498 574	447		546	602	280	830	510 583	*p* = 0.311

Bold indicate statistically significant difference *p* < 0.05

### Voluntary Coughs Versus Throat Clearings

The median duration of a voluntary throat clearing was longer than the median duration of a voluntary cough (*p* < 0.001) and the intensity in dB of the throat clearing was lower (*p* < 0.001) (Tables 1, 2, 3, 4). Regardless of the microphone type, the typical curvature of the amplitude contours of voluntary coughs (convex) and throat clearings (concave) were inverted (*p* < 0.001) with regard to each other showing that a throat clearing starts with a lower relative spectral energy (Figs. 5 and 6). A lower median sample entropy and a lower median kurtosis were also observed for throat clearings (*p* < 0.001). However, the relative energies observed in the spectral bands 0–400 Hz and 400–800 Hz were comparable to the relative energies in voluntary coughs with the skin-contact microphone (Table 4).

### Voluntary Coughs Versus Induced Reflexive Coughs (Skin-Contact Microphone Only)

The cough length and the spectral relative energy below 800 Hz were not statistically significantly different between voluntary and reflexive coughs (Tables 7–8). The convexity of the curvature of the amplitude and kurtosis contours were larger for reflexive than for voluntary coughs (*p* < 0.001 and *p* = 0.002, respectively). Visual differences were the most prominent at the onsets (higher kurtosis and shorter pulse) and offsets (higher kurtosis) of the reflexive cough signals (Fig. 7). The median of the sample entropy contour and the relative energy found above 800 Hz were also higher for reflexive than for voluntary coughs (*p* < 0.001).

## Discussion

The aim of our study was to explore voluntary cough, throat clearing, and induced reflexive cough sounds as possible acoustic markers of dysphagia and aspiration. Because this topic is underresearched, this article focuses on assessing acoustical differences between voluntary and reflexive coughs as well as throat clearings in a healthy population.

The cough analysis developed for the purpose of our project enabled us to extract temporal and spectral acoustic cough features. Temporal: cough length, cough intensity in dB and contours of cough amplitude, sample entropy, and kurtosis. Spectral: the relative energy in frequency bands < 400 Hz, 400–800 Hz, 800–1600 Hz, 1600–3200 Hz, and > 3200 Hz as well as the spectral centroid in Hz.

Analyses of voluntary coughs suggested that the conventional three-phase sequential pattern of a cough sound—burst-like onset, frication noise owing to turbulent airflow, voicing—is idealized because the three sub-segments must not be present for all subjects and also because burst, turbulence noise, or voicing may be superimposed. The spectral decomposition suggests that a cough sound is a transient signal with a possible coexistence of frication noise—presumably due to the airflow passing through the glottis and striking the supraglottic structures—and lower frequency noise—presumably generated by the airflow modulated by the vibrations of the vocal folds, false vocal cords, and/or aryepiglottic folds [46, 47]. Therefore, acoustic cough emissions are evocative of hard-onset growls rather than of forceful glottal stops. Indeed, the latter involve the true vocal folds exclusively [27], whereas the former also implicate the adduction/abduction and vibration of the false vocal cords as well as the aryepiglottic folds [48].

The comparison of the voluntary cough signals recorded by the two transducers showed that the skin-contact microphone underreports high-frequency components [49]. This observation may be explained by the attenuation of the acoustic signal propagating through the tissue of the neck before the signal is recorded by the skin-contact microphone, but also by the lack of the boosting of the high-frequency components (high-pass filtering) of the cough signal owing to the acoustic radiation at the lips.

We also performed analyses of voluntary throat clearings. We decided to include this airway clearance maneuver because its perceptual assessment is common during a clinical swallowing evaluation, but it is poorly documented in the literature. In addition, the perceptual detection and definition of a throat clearing are reported inconsistently [18].

Our analysis showed that a throat clearing starts with a weaker onset pulse (concave curvature of the amplitude contour, lower average, and slope as well as lower convex curvature of the kurtosis contour) compared to a voluntary cough and that it is characterized by one main fragment composed of oscillations all along the signal. These observations corroborate the anatomical differences observed while producing a voluntary cough versus a throat clearing. Indeed, the latter implies a partial vocal fold closure only, but which is steady all along the signal [18, 50]. Conversely, a voluntary cough involves a complete glottal closure before release [11]. Also, a throat clearing does not have an inspiratory phase, in contrary to a voluntary cough [1]. Besides, Xiao et al. examined the manometric profiles of both coughing and throat clearing [51]. The authors observed a greater number of repetitive pressurizations and a more vigorous upper esophageal sphincter contraction for coughing compared to throat clearing. One may therefore assume that, because of the lack of complete vocal closure, the lack of prior inspiration and the lower pressurization during a throat clearing, the acoustic relative energy at the onset is decreased. Hence, the difficulty in detecting and distinguishing auditorily both of these protective maneuvers may be avoided by focusing on the distinctive objective acoustic features mentioned above. As observed for voluntary coughs, a lower relative energy was observed in higher frequency bands when the throat clearing signal was recorded with the skin-contact microphone owing to the attenuation of high-frequency noise propagating through the neck tissue.

We also examined induced reflexive coughs. The literature reports that airflow features of reflexive coughs (induced by a tussigen) are reliable markers of cough effectiveness preventing aspiration in patients with dysphagia [17, 52, 53]. Induced reflexive coughs were recorded by means of two procedures: the urge-to-cough and the suppressed reflexive cough methods. The urge-to-cough method is considered to enable estimating the natural tussigen threshold, but it may be modulated by cortical expectations of cough occurrence [44]. The identification of the natural cough threshold is valuable in the context of dysphagia because it reports the biological perceived need to cough [54]. The suppressed reflexive cough method, which reports the dose at which participants can no longer voluntarily control their cough response, involves stronger cortical control and inhibition [55]. Mills et al. have demonstrated that participants may fail producing a suppressed reflexive cough (i.e., they do not cough regardless of the tussigen dose) [56]. We also observed a lack of cough response in 20% of the participants with the suppressed reflexive cough method. Therefore, the combination of both methods might be useful to increase the chances of recording induced reflexive coughs with at least one method. Our analysis did not show any statistically significant difference between temporal and spectral acoustic features obtained via the urge-to-cough and the suppressed reflexive cough methods. Therefore, we grouped induced reflexive cough samples obtained via both methods.

We observed that an induced reflexive cough starts with a stronger and briefer onset pulse (higher convex curvature of the amplitude contour (*p* < 0.001) and higher average (*p* < 0.001), steeper slope (*p* = 0.008), and higher convex curvature of the kurtosis contour (*p* = 0.002)) compared to a voluntary cough. The onset of a reflexive cough was also followed by statistically significant higher values of the average (*p* < 0.001) and concave curvature (*p* < 0.001) of the sample entropy contour and higher spectral energy above 800 Hz (*p* < 0.001). This observation suggests that the release of reflexive coughs is more forceful and sudden. This supports findings by Lasserson et al. reporting physiological distinctions between voluntary and reflexive coughs [57]. Indeed, they found that during a voluntary cough, the level of activation of expiratory and accessory muscles is sequential and can be modulated depending on the need perceived. In contrast, for reflexive coughs, these muscles are activated simultaneously without voluntary regulation.

We observed acoustic differences between the first and second reflexive cough signals (cough #1 and cough #2). Indeed, the concavity of the sample entropy contour of cough #1 was statistically significantly more marked compared to cough #2 (*p* = 0.037). This observation suggests that differences exist between cough #1 and cough #2 with regard to subglottal pressure and/or abruptness of the release. This interpretation is confirmed by statistically significantly steeper slopes of the amplitude (*p* = 0.006) and kurtosis (*p* = 0.008) contours and higher relative energy in the lower frequency band 400–800 Hz in cough #2 compared to cough #1 (*p* = 0.019). One possible explanation is that a weaker onset burst and weaker oscillations in cough #1 may reflect a weaker glottal closure directly after the tussigen inhalation.

The acoustic analyses carried out show that a voluntary cough, a voluntary throat clearing, and a reflexive cough are acoustically significantly distinguishable and that they report complementary information. These observations will have to be taken into account for the assessment of these maneuver-related acoustic features as possible markers of aspiration in HNC patients with RAD.

## Limitations

There are possible limitations of our study. Asking the participants to produce both throat clearings and voluntary coughs without an agreed upon definition or distinction being available may have been confusing. In addition, providing a demonstration to the participants who mixed up both maneuvers may also have influenced the signals produced. This limitation might have been avoided by providing a demonstration from the start to all participants.

A second limitation is the impossibility to measure intensity in dB for induced reflexive coughs. Indeed, the anesthesia face mask attenuates and filters the acoustic cough signal. In addition, the amplitude of the acoustic cough signal does not report intensity in dB because it is influenced by the pre-amplifier gain and the microphone position.

Another limitation is the under-emphasis of high-frequency bands by the skin-contact microphone, which therefore underreports the acoustic energy ascribed to turbulence noise propagating through the neck tissue compared to cough sounds acoustically radiated at the lips.

## Conclusion

The objective cough sound analysis developed for the purpose of our project has enabled us to extract temporal and spectral acoustic features of three protective maneuvers: voluntary coughs, throat clearings, and induced reflexive coughs.

Voluntary and reflexive coughs are composed of two or three fragments. The first fragment is characterized by a strong onset pulse and oscillations. The second fragment is characterized by stable frication noise. The potential third fragment is characterized by mechanical oscillations when a voiced coda is part of the cough signal.

A throat clearing sound is composed of one main fragment characterized by large low-frequency oscillations and feeble frication noise.

Acoustic features describing voluntary cough sounds, voluntary throat clearing sounds, and induced reflexive cough sounds are significantly statistically distinguishable.

We would recommend to take into consideration acoustic features related to voluntary coughs, throat clearings, and reflexive coughs for further work exploring airway protective abilities. Work is currently underway to identify relevant acoustic cough and throat clearing features as possible markers of dysphagia and aspiration in head and neck cancer patients with radiation-associated dysphagia.

### Clinical Implications

Radiation-associated dysphagia in HNC patients following (chemo) radiotherapy may involve ineffective or absent coughing. Coughing is assessed auditorily during a clinical swallowing examination. However, low inter- and intra-rater reliability regarding the auditory assessment of cough is reported. Therefore, subjective scoring of coughing may be considered as an unreliable predictor of the risk of penetration and aspiration. This suggests a need for innovative methods obtaining objective markers of cough efficacy in a natural setting.

Our research group is currently exploring the analysis of cough sounds as an alternative assessment method in HNC patients with RAD. Such a method would be implementable in daily life because it is based on software running on widely available devices and a skin-contact microphone. The software developed for the purpose of this research analyzes transient signals automatically, objectively, and consistently. In addition, the skin-contact microphone used in this study does not disturb the food intake and does not record external environmental noise. Finally, this equipment (microphone and software combined) is non-invasive, low cost, and suitable for a conventional clinical swallowing evaluation, during a meal or during a Fiberoptic Endoscopic Evaluation of Swallowing.

## Data Availability

The datasets generated during the current study are not publicly available since they will contain patient data and the informed consent does not include sharing-data publicly. The datasets are available from the corresponding author on reasonable request.
